# Visual Assessment of Blood Plasma versus Optical Transmittance and Refractive Index Measurements for Quantifying Lipemia

**DOI:** 10.3390/diagnostics12020510

**Published:** 2022-02-16

**Authors:** Roberto Márquez-Islas, Argelia Pérez-Pacheco, Rosa Quispe-Siccha, Laura Beatriz Salazar-Nieva, Augusto García-Valenzuela

**Affiliations:** 1 Unidad de Investigación y Desarrollo Tecnológico, Hospital General de México Dr. Eduardo Liceaga, Dr. Balmis No.148, Colonia Doctores, Cuauhtémoc, Mexico City 06726, Mexico; argeliapp@ciencias.unam.mx (A.P.-P.); rosa.quispe@gmail.com (R.Q.-S.); 2 Laboratorio de Química de Radiaciones, Instituto de Ciencias Nucleares, Universidad Nacional Autónoma de México, Circuito Exterior s/n, Ciudad Universitaria, Coyoacán, Mexico City 04510, Mexico; laurasn@ciencias.unam.mx; 3 Instituto de Ciencias Aplicadas y Tecnología, Universidad Nacional Autónoma de México, Coyoacán, Mexico City 04510, Mexico; augusto.garcia@icat.unam.mx

**Keywords:** lipemic human plasma, plasma refractive index, transmittance measurements

## Abstract

Today, visual classification of the degree of lipemia in blood samples is frequently performed in clinical laboratories. However, achieving standardization of this classification at low cost and with fewer resources is an objective that is still under development. In this work, a comparison is made between the visual inspection and optical measurements of blood plasma for quantifying lipemia. The plasma refractive index’s real part was measured using an Abbe refractometer and transmittance measurements were made at a 589 nm wavelength and wavelengths ranging from 320 to 1100 nm in the spectral region, respectively. Taking the slope of the transmittance spectrum at two specific wavelengths, it is possible to establish a more standardized selection criterion and implement it quickly using low-cost optical devices. Furthermore, using the proposed transmittance-spectrum-slope method, statistically significant differences (*p* < 0.05) were found between healthy blood samples and lipemia 1, 2, 3, and 4. However, there were no statistical differences between lipemia 1 and 2.

## 1. Introduction

Blood donation is a fundamental resource for the adequate treatment of patients in a hospital. Blood banks continually receive voluntary donors to maintain a blood supply that satisfies the hospital’s needs and enables medical processes. The main activity of the blood bank is to establish and enforce criteria for the selection of donors and guarantee that the blood and its derivatives (platelet concentrates, plasma, albumin) [1] are processed and subsequently used in the treatment of hospital patients. In addition, serology studies are carried out on the blood of voluntary donors to guarantee that there is no risk of transmission of any disease (hepatitis, syphilis, HIV, malaria) [2]. Currently, there is a need to increase the number of blood units worldwide because its use is not exclusive to transfusions. Some blood products, such as platelet concentrates, are being investigated for use in maxillary surgeries [3], and transfusion of packed red blood cells (PRBCs) is being explored for treating anemia in critically ill patients [4]. However, the risk of contracting a transfusion-transmitted infection remains. Hence, one of the main concerns of blood banks is to develop techniques to process and eliminate potential pathogens from donated blood through chemical procedures, ultraviolet light, or visible light for the inactivation of pathogens and significantly decrease the threat of pathogen transmission through plasma, platelets, and other blood derivatives [5,6]. However, these regulations are not sufficient since some infectious agents of pathologies require specific detection devices. Therefore, easily accessible and low-cost tests and devices must enable a more efficient preselection of volunteers and reduce each analysis’s cost. Consequently, it is essential to preselect samples from donors to ensure that the blood collected meets the established quality standards.

A confidential interview, a comprehensive physical test, and a questionnaire are applied to establish the medical history of donors to determine their health status. In addition, in some countries, a pre-donation lipemia test is requested. These are, in general, the initial steps that the blood bank implements for potential donors. If the volunteer meets all of the requirements specified in the preselection procedure, the donation process can start. Otherwise, the volunteer is excluded for a certain period or permanently depending on the result of the interview. Donated blood, drawn by venipuncture from the arm, is placed into a tube with an anticoagulant as the first step. The sample is then examined and centrifuged to separate the plasma from the other components. This plasma is observed with the naked eye by a technician, who evaluates it for the presence of pathologies, such as lipemia, icterus, or hemolysis [7,8]; if any of these pathologies are found, the sample is discarded since they can mask the presence of other diseases [9].

Among the errors in a visual assessment are those incurred in the classification of hemolyzed blood samples, where up to 50% are rejected. Using an automated method, 35% of those samples would be viable for use [10]. In the case of the identification of samples with lipemia, the percentage of discarded samples that could be used varies from 5% to 12% [11], and for icterus, it has been estimated at 1.5% [10,12]. In this study, we focused on the quantification of lipemia.

Lipemia is a consequence of the accumulation of lipid particles in the blood plasma, commonly in the form of chylomicron particles with various sizes from 70 to 100 nm and particles of “very-low-density lipoproteins” (VLDLs) with sizes from 35 to 200 nm. VLDLs are mainly responsible for the cloudy appearance of plasma. In many cases, sample classification is carried out visually based on the turbidity of the plasma, which is one of the initial criteria for rejecting the donor sample. Different laboratory techniques have proved that visual detection is not appropriate for determining the different degrees of lipemia present in blood samples [13]. The use of automated instruments offers a more objective methodology to obtain consistent, reproducible, and rapid results [14,15]. However, this automated analysis necessitates the use of clinical laboratory elements, high costs, and time. Therefore, it is necessary to provide support tools to professionals who select dozens of blood samples per day. Developing a method that allows the accurate classification of lipemia in venous blood samples can increase the number of accepted donors. This would help prevent false positives that result from visual observation and guide the development of a device that helps to establish a criterion for the classification of blood samples and serves as a reference for the training of members who join the blood bank. Therefore, in this work, we investigated the potential of two simple optical measurements to detect and classify lipemia, namely, the transmittance spectra and refractive indices of plasma from volunteers’ blood. This analysis can be extended to identify and quantify other types of interferences or pathologies present in blood or other biofluids.

## 2. Materials and Methods

Plasma samples of blood from voluntary donors who came to the blood bank of Hospital General de México “Dr. Eduardo Liceaga” (HGMEL) were studied. Each sample is classified as healthy or with some degree of lipemia according to the specialist’s criteria. All samples are stored in a 4 mL vacutainer tube and are centrifuged at 3500 rpm for 15 min. The plasma sample resulting from centrifugation is mainly composed of water (90–92%). The remaining plasma percentage is primarily constituted by plasma proteins grouped into three large groups: albumin, globulins, and fibrinogens. Albumin is the main protein in blood (60%) and responsible for regulating the total plasma volume [16] and lipid particles, especially triglycerides, which give the plasma sample a turbid, milky appearance. The triglyceride particle size varies from 6 to 1000 nm. The largest particles are chylomicrons (70–1000 nm), which are potentially responsible for producing turbidity in the sample [17] and can be visually detected if the triglyceride concentration is higher than 3.4 mmol/L [18]. Once the components have been separated, the laboratory personnel conduct a visual assessment, qualifying the color and the turbidity level that the plasma exhibits. The “−” mark is assigned to samples that show an amber-colored plasma without any trace of turbidity (healthy sample), while 1–4 cross symbols (from “+” to “++++”) are assigned to classify lipemic samples according to their turbidity level (from lowest to highest), assigned by an expert, following the standard established by the blood bank itself. However, these criteria may vary between different blood banks.

**Figure 1:**
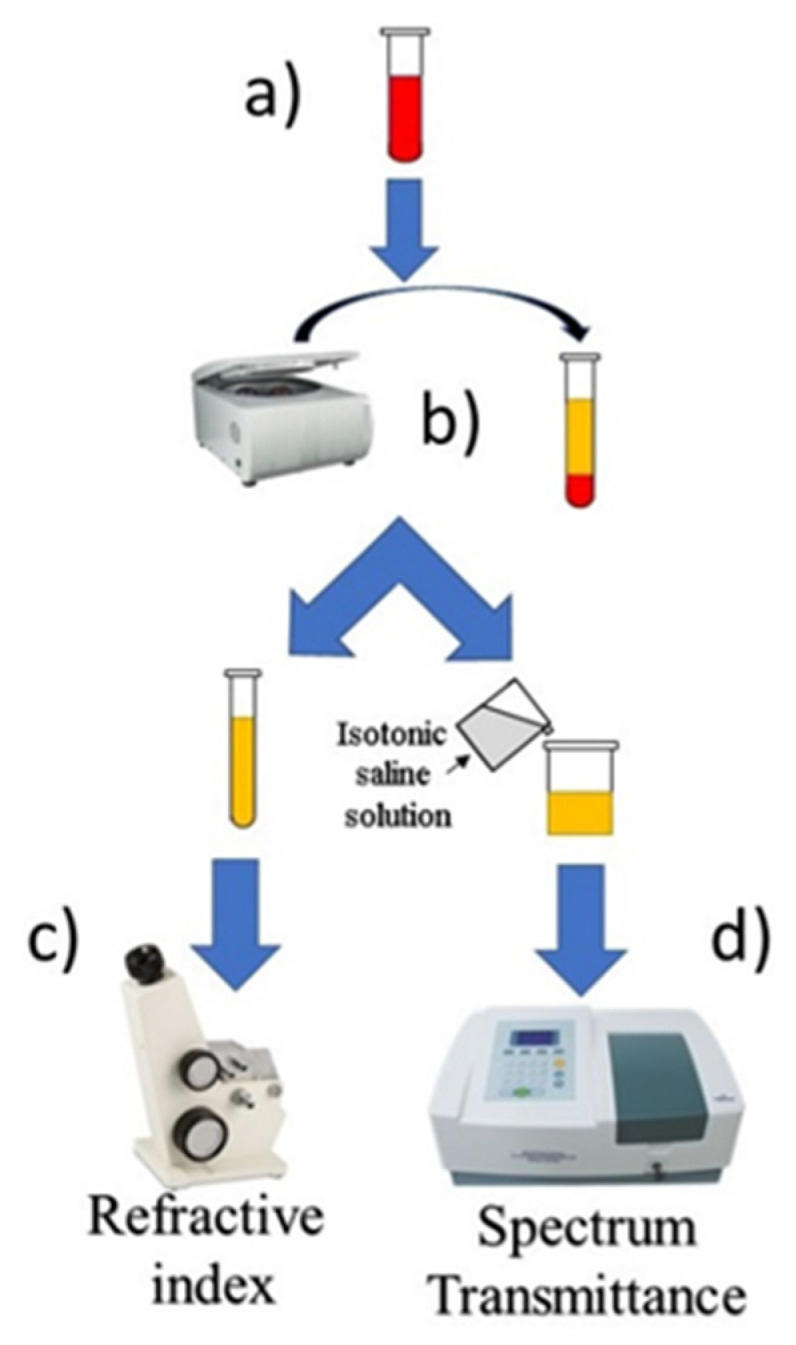
Methodology for obtaining the plasma refractive index and transmittance spectrum. Whole blood sample for analysis (**a**). Centrifugation to separate the blood sample into its components (**b**). The samples are divided into two volumes: one sample is measured using the Abbe refractometer (CJHZYG, model 2WAJ, Shenzhen, China), and its RI is measured (**c**); the other sample is diluted with a 0.9% NaCl solution, and its absorbance and transmittance spectrum is measured using a spectrophotometer (XZBELEC, model V1710, Shenzhen, China) (**d**).

We analyzed the plasma samples’ transmittance spectra and refractive indices as an alternative means to classify the degree of lipemia (scheme in Figure 1) and compared the results with the visual method. Our methodology for sample analysis can be summarized in the following steps, which are illustrated in Figure 1.

The samples were centrifuged following the standard method to separate erythrocytes and leukocytes from plasma. Once the constituents of the blood sample were separated, a small plasma volume was taken to measure the refractive index (RI) directly using an Abbe refractometer. Then, another volume was prepared to measure the transmittance and absorption spectrum in the spectrophotometer (model V1710). For transmittance measurements with the spectrophotometer, isotonic solutions (IS) (0.9% NaCl) were used to obtain a baseline in the wavelength range from 320 nm to 1100 nm. In order to prevent saturating the spectrum and achieve a good transmittance signal, the lipemic plasma samples were diluted with IS in a proportion of 40–60%, respectively; this dilution was performed because the undiluted sample marked with “++++” completely extinguished the transmittance spectrum.

## 3. Results

**Figure 2:**
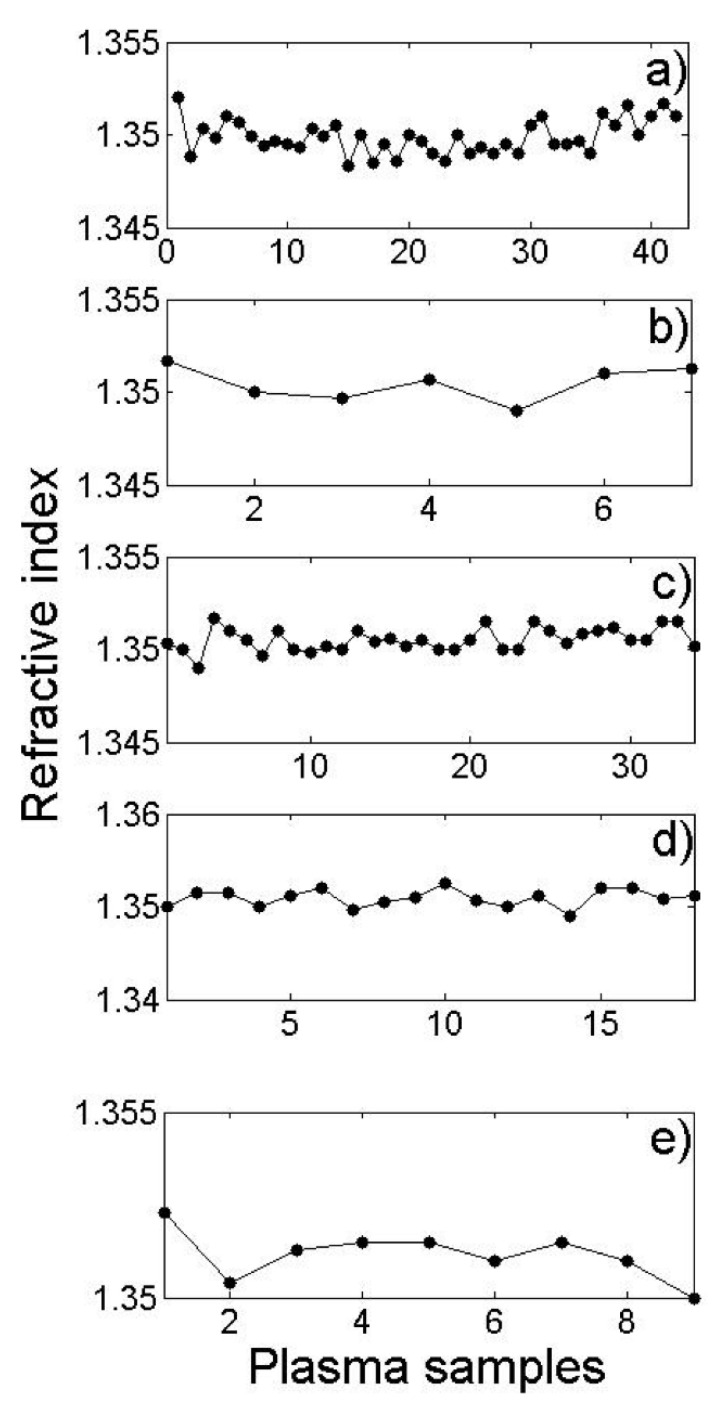
Graph of the plasma samples’ refractive indices measured with the Abbe refractometer: (**a**) healthy samples, (**b**) lipemia 1, (**c**) lipemia 2, (**d**) lipemia 3, (**e**) lipemia 4.

The average RIs measured with the Abbe refractometer are shown in Table 1. They are grouped according to the classification designated for the laboratory expert: lipemia 1 (L1), lipemia 2 (L2), lipemia 3 (L3), and lipemia 4 (L4). These are plotted in the graph in Figure 2. Each dot represents the plasma RI of a single volunteer.

Figure 2 shows a higher frequency of healthy donors, followed by L2, L3, and L4. It is worth clarifying that some samples were excluded because some volunteers did not follow the fasting requirements (from 8 to 12 h before donating with free access to water). According to the measurement results obtained with a standard Abbe refractometer, the RI of the plasma is not a sensitive enough measurement to determine the degree of lipemia since lipid particles and triglycerides at the corresponding concentrations do not significantly affect the real part of the IR.

**Figure 3:**
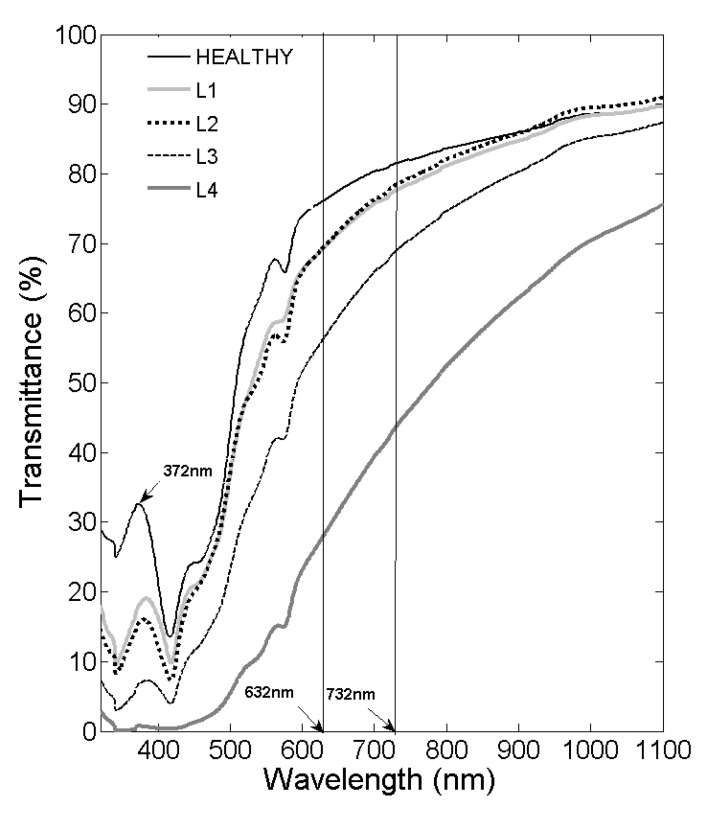
Transmittance spectra (average value) of healthy and lipemic blood samples classified from L1 to L4 at wavelengths between 320 nm and 1100 nm. The graph indicates the location of the two wavelengths that are used to calculate the slope between the transmittance values 632 nm and 735 nm.

Figure 3 shows the average transmittance spectra of the plasma samples according to the degree of lipemia. In the 330–1100 nm range, the transmittance spectrum is affected by absorption peaks caused by hemoglobin (usually, tiny traces remain due to hemolysis of some erythrocytes). The largest peak is located at 420 nm, and the other smaller ones are at 541 nm and 576 nm. Icterus manifests as a broad absorbance peak around 460 nm, which sometimes interferes with other absorbance peaks due to hemoglobin. In lipemia, attenuation of collimated light occurs in a wide range of wavelengths from UV to visible, slowly decreasing with the wavelength [19]. Strong water absorption affects the transmittance at lengths close to infrared (IR) and far IR. The spectra of healthy samples overlap with lipemia 1 and 2 at wavelengths between 800 nm and 1100 nm. At wavelengths between 650 nm and 800 nm, hemolysis and icterus do not interfere in the transmittance spectrum, and there are no characteristic absorption peaks of any other trace molecule. Light is attenuated only due to the scattering of lipoprotein particles.

### 3.1. Average Slope between Selected Wavelengths

As shown in Figure 3, in the average spectrum of healthy plasma samples in the wavelength interval from 600 nm to 780 nm, a lower extinction of the spectrum is observed due to the low concentration of lipids, and a peak is also observed with very marked transmittance at 372 nm. Conversely, samples with degrees of lipemia from 1 to 4 extinguish very markedly in this wavelength interval and exhibit a peak in transmittance that has shifted from 372 nm to 382–384 nm. The spectra of the samples with lipemia exhibit more pronounced extinction due to the higher concentration of lipid particles that scatter light compared to healthy samples’ spectra. These characteristic shapes of transmittance spectra can be used to propose a method for quantifying lipids; we should note that the shape of the spectrum is independent of the intensity of the transmittance.

We propose a calculation to quantify the lipid concentration in plasma using the value of the slope of the line that joins the transmittance values at 632 nm and 732 nm wavelengths. Furthermore, the proposed wavelengths are very accessible in a device using LEDs, which are very stable and low-noise sources of light. In this wavelength interval, the only effect on the spectrum is the concentration of lipids in the plasma. The slope of a line is an essential geometric concept, which we can interpret as a measure of the inclination of a line and indicates the amount by which the value of the variable “y” (transmittance) increases or decreases when the “x” variable (wavelength) increases by one unit. At a higher concentration of lipids, the spectrum’s extinction becomes more significant. Therefore, the slope of the line that joins the transmittance values at the two proposed wavelengths will be greater. The calculation of the slope is carried out as follows:

(1) $$\mathrm{slope}=\frac{T_{2}\left(\lambda_{732}\right)-T_{1}(\lambda_{632})}{\lambda_{732}-\lambda_{632}}\;\;\;\;$$

where T_1_ and T_2_ are the transmittance values at wavelengths (λ) of 732 nm and 632 nm, respectively. We calculated the slope of the line that joins the transmittance values at the two established wavelengths. Subsequently, we grouped the results of the technician’s classification of the samples before donation.

### 3.2. Statistical Analysis

**Figure 4:**
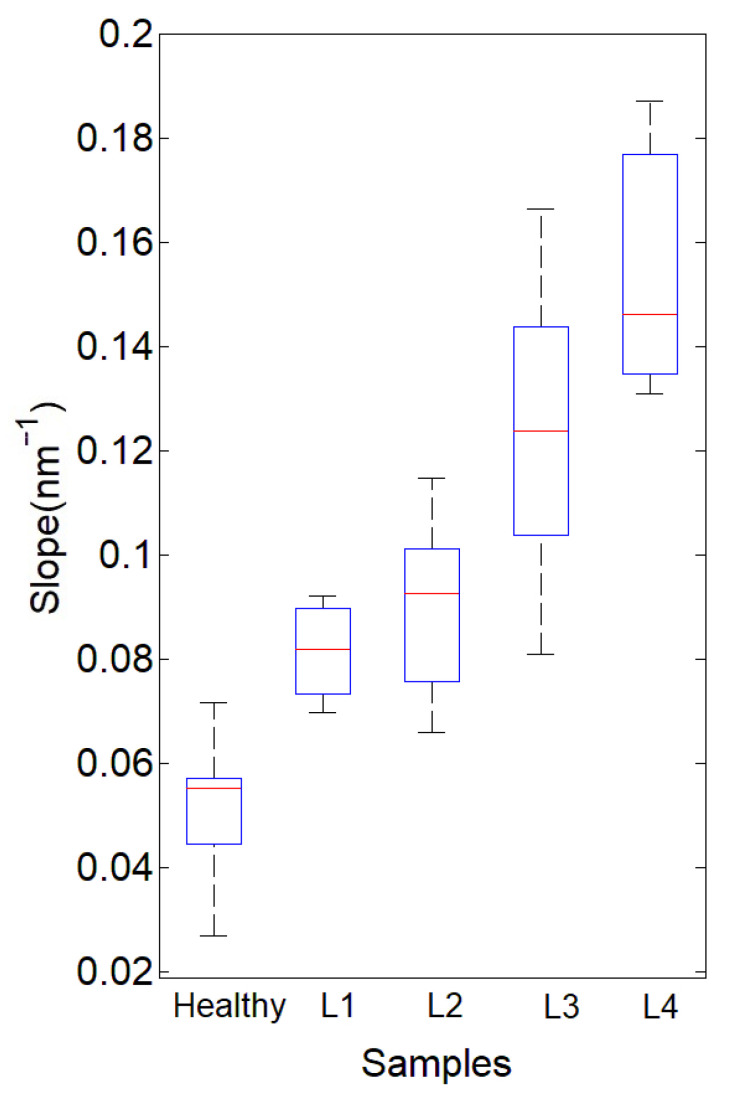
Boxplot and whisker plot of the slope data. Healthy samples, L1, L2, L3, and L4.

The SPSS v. 21 statistical program was used for data analysis. The sampled data from the population did not follow a normal distribution. To analyze the differences between two independent samples, the nonparametric Mann–Whitney U test was used. Values with *p* ≤ 0.05 were established as significant. Figure 4 is a boxplot depicting the average of the slope data obtained for each group. The median values in the healthy, L1, L2, L3, and L4 groups were 0.52 ± 0.01, 0.81 ± 0.01, 0.88 ± 0.01, 0.12 ± 0.02, 0.15 ± 0.02, and 0.80 ± 0.03, respectively. The analysis shows statistically significant differences between all groups except L1 and L2, with 95% confidence.

## 4. Discussion

The slope value between the transmittance points at two wavelengths was calculated for all spectra obtained from healthy and lipemic plasma samples using Equation (1). In Figure 4, we present boxplots depicting the average of the slope data obtained for each lipemia classification group given in the first part of this work. The red lines show the slope’s median value in each plasma group.

The calculated slopes of the L1 and L2 samples are dispersed in a range of values between 0.066 nm^−1^ and 0.114 nm^−1^. We can see that, as we expected, the slope values of healthy samples are the lowest among all groups. In the second quartile below the mean, we find that the dispersion of the data is very high, and in contrast, the dispersion of data in the third quartile is markedly lower. More than 75% of the values of the slopes of healthy samples are below 0.06 nm^−1^, and only in four cases are they in the fourth quartile (15%). In addition, some of the values in the lower quartile of L3 are within the range of L1 and L2. What should be highlighted is that according to our proposed methodology, the healthy samples are clearly differentiable from the L1 samples. The adjacent values (whiskers) of the healthy samples are farther from the median but are not out of the range, and values that overlap with L1 and L2 account for 11%. The L3 and L4 samples show a range of slope values between 0.08 nm^−1^ and 0.19 nm^−1^. However, it is possible to establish the difference between healthy samples and L3 to L4 samples. The boxplot of L1 and L2 overlap and extend beyond both medians, which indicates that the transmittance spectra method and the visual assessment method do not coincide in distinguishing lipemia of the first and second degrees. We must consider that the expert’s visual classification may fail to quantify the degree of lipemia in the selected samples. The possibility of important errors is always present in this evaluation, as it is subjective and less reliable compared to analytical turbidity evaluation [20], and thus, at the moment, it is not possible to say which method is more appropriate for L1 and L2 samples. The results obtained from the analysis of healthy and lipemic samples selected by visual evaluation show that there is an acceptable concordance with the estimate. Our method allows us to distinguish healthy from lipemic plasma. The probable error in selecting lipemic samples by the visual technique can be reduced if a less subjective auxiliary device is established, which could help ensure that healthy samples are not unnecessarily discarded in the preanalytical phase. Automated systems that determine the lipemic index can be highly efficient and obtain reproducible results [21], but that does not rule out false positives in plasmas without turbidity due to the presence of paraproteins [22]. The efficient selection of samples in the preanalytical phase is one of the important factors that can improve the use of resources in hospitals where the use of automated methods is not economically feasible.

## 5. Conclusions

This work compares the visual classification of the lipid concentration in blood samples with an optical methodology for rapid quantification. The proposed method entails measuring the plasma’s transmittance at two specific wavelengths (732 nm and 632 nm). From the results obtained, it is clear that the optical transmittance method can be used with a good degree of confidence to differentiate healthy samples from lipemic samples. However, at the moment, it does not allow the accurate classification of differences between L1 and L2 samples. Overall, there is a disagreement in 11% of cases between the visual assessment method and the two-wavelength optical transmittance method in differentiating healthy samples from lipemic ones. Thus, with a confidence of about 90%, the measurement of transmittance at two wavelengths can be used to differentiate healthy samples from lipemic samples of grades L1 or L2. The confidence increases to 100% when the lipemic grades are either L3 or L4. The optical transmittance option can support the classification of large volumes of samples. It can be used to standardize the process at low cost with a device that can be made with off-the-shelf elements, such as LEDs and regular silicon photodetectors.

## Figures and Tables

**Table 1:** Average refractive indices of plasma samples measured with the Abbe refractometer according to the visual classification of the degree of lipemia assigned by the blood bank technician. We can see that despite the visible differences in turbidity, the refractive index’s change starts from the third digit considering the uncertainty.

Sample/Classification	Refractive Index
Healthy	1.3498 ± 0.0022
L1	1.3504 ± 0.0014
L2	1.3505 ± 0.0013
L3	1.3509 ± 0.0019
L4	1.3511 ± 0.0012

## Data Availability

The data that support the findings of this article are available from the corresponding authors on reasonable request.
